# Medium-Term Effects of Increased Water Intake and Head-Up Sleep on Cardiovascular Health

**DOI:** 10.1016/j.jacadv.2024.101536

**Published:** 2025-01-08

**Authors:** Meihan Guo, David Montero

**Affiliations:** a Faculty of Medicine, Hong Kong University, Hong Kong, China; b CardioVascular Institute and Division of Cardiology, Department of Medicine, Harvard Medical School, Beth Israel Deaconess Medical Center, Boston, Massachusetts, USA; c Department of Medicine, School of Clinical Medicine, Hong Kong University, Hong Kong, China; d Libin Cardiovascular Institute of Alberta, University of Calgary, Calgary, Alberta, Canada

**Keywords:** cardiorespiratory fitness, cardiovascular function, female sex, head-up sleep, increased fluid intake, lean body mass

## Abstract

**Background:**

Whether medium-term increased water intake alone, or in combination with co-adjuvant nonexercise interventions aimed to expand blood volume (BV), improve the human cardiovascular phenotype and cardiorespiratory fitness remains unexplored.

**Objectives:**

The purpose of this study was to determine the medium-term impact of increased (+40%) fluid (water) intake (IFI) or IFI plus head-up sleep (IFI + HUS) on BV and the cardiovascular phenotype in healthy individuals.

**Methods:**

Healthy adults (n = 35, age 42 ± 18 years, 51% female) matched by sex, age, body composition, physical activity, and cardiorespiratory fitness were randomly allocated to IFI or IFI + HUS for 3 months. Body composition and BV were determined via DXA and indicator-dilution methods. Cardiac filling, output, and peak O_2_ consumption (VO_2peak_) were assessed via high-resolution echocardiography and pulmonary gas analyses at rest and during incremental exercise.

**Results:**

Intravascular volumes, comprising plasma and red blood cell volumes, were not modified by IFI or IFI + HUS. Cardiac volumes at rest, specifically left ventricular (LV) end-diastolic volume and stroke volume (SV), and systolic emptying rate were increased after IFI and IFI + HUS (*P* ≤ 0.007); the effects on SV and systolic emptying rate were larger in IFI + HUS vs IFI (*P* ≤ 0.037). Arterial elastance and cardiac afterload were similarly reduced by IFI and IFI + HUS (*P* ≤ 0.006). Moreover, resting LV diastolic filling rate and lateral wall e’ velocity were only increased after IFI + HUS (*P* ≤ 0.031). During exercise, neither SV, cardiac output, and peak VO_2_ were altered by IFI or IFI + HUS.

**Conclusions:**

Medium-term increased water intake largely expands the resting volume and output of the LV while reducing arterial elastance and cardiac afterload, without altering intravascular volumes, cardiac or aerobic capacities. With the addition of HUS, relaxation properties of the resting LV are further improved.

Despite the normal function of the human body requires the consumption of enough fluid, specifically water, the optimal amount remains uncertain. Remarkably, conflicting findings have been reported in observational studies regarding the relationship of increased water intake with all-cause mortality in the same study population.^1^^,^^2^ Moreover, the potential risks of excessive water intake, particularly during exercise, have been emphasized.^3^ However, thus far, longitudinal studies assessing the short-, medium- or long-term effect of increased water intake in healthy humans are surprisingly scarce and limited in their scope.^4^^,^^5^ Accordingly, the impact of chronically increased water intake on vital functions such as those accomplished by the cardiovascular and respiratory systems has yet to be determined.

The main function of the cardiovascular system, that is, to circulate blood, primarily depends on the filling of the heart.^6^ In healthy humans, cardiac filling is not maximized, that is, there is an unused reserve to enhance the filling and output of the ventricles.^7^ Such a reserve can be accessed via increases in blood volume (BV).^7^ Since the predominant component of BV is water, enhancing its intake may appear to be a straightforward approach to augment BV, cardiac filling, and output. Yet, body fluids, notably the intravascular volumes, are tightly regulated by the endocrine system.^8^ Any *excess* in water intake may be excreted in the urine, keeping BV stable, unless a modification occurs in the endocrine axes and specific hormones that regulate BV—renin-angiotensin-aldosterone system, natriuretic peptides, and vasopressin.^9^ In this regard, an established intervention to modify BV-regulating hormones in the direction of fluid retention is head-up tilt.^9^ Since the 1940s, head-up sleep (HUS) has been recommended as a lifestyle intervention to treat orthostatic intolerance, with the underlying rationale of improving hemodynamic stability via increased BV and cardiac filling.^10^ Prolonged moderate HUS (≥3 months, +10° elevation of the head of the bed) has been shown to increase BV and orthostatic tolerance in patients with vasovagal syncope.^11^ Nonetheless, the impact of medium- and long-term HUS in the healthy population has never been assessed, plausibly due to the logistical and partner-related challenges associated with this intervention.^12^ In the absence of evidence, in principle, the combination of increased water intake and HUS might have the potential to elicit substantial intravascular and cardiac volumetric adaptations enhancing the capacity to circulate blood and deliver O_2_ to the tissues.

Accordingly, the purpose of this study was to originally determine the medium-term (3-month) impact of increased fluid (water) intake (IFI) or IFI plus HUS (IFI + HUS) on BV and the cardiovascular phenotype, pulmonary O_2_ extraction and uptake at rest and during incremental exercise up to peak effort, in healthy individuals. We hypothesized a greater effect of IFI + HUS vs IFI on intravascular and cardiac volumetric expansion and capacity to deliver and consume O_2_.

## Methods

### Study participants

Forty healthy women and men throughout the adult lifespan (range 18-68 years) were randomly allocated to IFI or IFI + HUS interventions lasting 3 months, using covariate adaptive randomization conforming to sex, age, and physical activity. Exercise training history and moderate-to-vigorous physical activity (total and specific to endurance exercise) over participant’s lifetime as well as thoroughly detailed during the last 3 months prior to the study were assessed at screening, as previously described.^13^ Inclusion criteria comprised healthy status according to health/clinical questionnaires and resting echocardiography/electrocardiogram screening, absence of current medical symptoms and medication, and no history of disease. The study was approved by the Institutional Review Board of the University of Hong Kong and the Hospital Authority West Cluster (UW 21-401) and conducted in accordance with the Declaration of Helsinki. Prior to the start of the experiments, informed oral and written consents were obtained from the participants.

### Study design

Participants were required to report twice to our laboratory for testing (preintervention and postintervention). They were instructed to avoid strenuous exercise, alcohol, and caffeine 24 hours prior to their visit to the laboratory, as well as to maintain their usual physical activity and daily lifestyle habits except for the specific modifications required by the interventions (IFI and/or HUS, as detailed below), throughout the study period. All measurements were performed after a fasting period (≥4 hours) to avoid postprandial hemodynamic alterations.^14^ Time of day of testing sessions was kept constant for each participant. According to previous studies, the menstrual phase in women was noted but not fixed for testing as it does not influence the main study outcomes.^15^, ^16^, ^17^ Prior to starting the measurements, the participants completed health and clinical questionnaires additionally comprising nutritional information and rested in supine position for 20 minutes in order to stabilize cardiovascular and hematological variables.

In both IFI and IFI + HUS interventions, daily water and nonalcoholic fluid intake was settled to 42 mL kg^−1^, equivalent to ⁓2 to 3 L in normal weight adults (⁓40% increment over population-specific values).^18^ To this end, individuals were instructed on how to measure water intake and reach the required daily level, while keeping a diary during the intervention noting and specifying all types of fluid intake. In addition, individuals in the IFI + HUS intervention were subjected to HUS via adjustable bed risers (Shepherd Hardware) or 40-cm tall seats (Kyorigin) placed under the frame at the top of the bed, in order to position the entire body at a moderate (15°) angle during sleeping at night, which was regularly checked by the investigators. A heart rate (HR) monitor (Polar H10) was provided to participants in the IFI + HUS intervention, who were instructed to wear it during the night and keep a diary during the intervention noting any type of sleep alteration. Weekly communication was maintained between the investigators and the participants to ensure the normal development of the interventions. Individuals not fulfilling their expected fluid intake and/or HUS protocol during at least 90% of intervention days were excluded from the study.

### Measurements

The description of measurements (cardiac structure/function, aerobic capacity, intravascular volumes, and aerobic capacity) is provided in the Supplemental Appendix.

### Statistical analysis

Statistical analyses were conducted via SPSS, 26.0 (SPSS Inc). The between-group difference in the effect of the intervention on BV was the primary outcome. According to power analyses based on previous measurements performed with the same methodology,^19^ a total sample size of 34 participants provided 85% power to detect a clinically meaningful 5% between-group difference (IFI + HUS vs IFI) in BV (G∗Power v. 3.1.9.6.). The required sample size was calculated with the following variables: α (0.05), power (0.85), and effect size (1.07), the latter determined from the algorithm recommended by Cochrane guidelines to estimate the SD of the change, using the hematological data previously reported.^19^^,^^20^ Based on preliminary testing and previous medium-term lifestyle interventions in our laboratory in similar populations, we estimated a dropout rate ≤20% for both interventions. Due to the logistical challenge intrinsic to the IFI + HUS intervention, an unequal randomization ratio was used for the allocation of participants to the interventions in order to ensure high statistical power.^21^ The statistical validity was confirmed by the normal distribution, determined with the Kolmogorov-Smirnov test, and the homogeneity of variances, assessed with the Levene’s test. Baseline characteristics were compared between groups via independent sample *t*-tests or Mann-Whitney *U* tests in the presence of not-normal distribution. The between-group comparison of the effect of the intervention on baseline variables was assessed with 2-way analysis of covariance, with group (IFI, IFI + HUS) as the between-subject factor, postintervention values as dependent variables and preintervention values as covariates. During incremental exercise, 2-way ANOVA with repeated measures was performed separately in each group to assess the effect of the intervention, with time (preintervention, postintervention) and exercise intensity (60, 70, 80, 90, and 100% of peak HR [HR_peak_]) as within-subject factors. The between-group comparison of the effect of the intervention (Δ, postintervention minus preintervention) was determined via 2-way ANOVA with repeated measures with group (IFI, IFI + HUS) and exercise intensity (60, 70, 80, 90, and 100% HR_peak_) as between- and within-subject factors, respectively. Post hoc comparisons at each exercise intensity were conducted if *P* was below 0.1 in the ANOVA. A 2-tailed *P* value <0.05 was considered significant. All data were reported as mean ± SD unless otherwise stated.

## Results

### General characteristics and intervention compliance

Table 1 presents baseline characteristics comprising demographics, anthropometrics, physical activity, and body composition. Age and sex (% female) were closely matched between IFI and IFI + HUS groups (*P* ≥ 0.725). Similarly, all anthropometrical variables (height, weight, body mass index, body surface area) were matched between groups (*P* ≥ 0.726). All individuals were nonsmokers and nonobese (body mass index <30 kg m^−2^). Physical activity, estimated by moderate-to-vigorous physical activity in total and specific to endurance exercise, did not differ between groups (*P* ≥ 0.177). Cardiorespiratory fitness, determined by peak O_2_ consumption (VO_2peak_) relative to kg of body weight or lean body mass, was matched between groups (*P* ≥ 0.445). Likewise, IFI and IFI + HUS groups presented similar body composition (bone mineral content, lean body mass, fat) (*P* ≥ 0.283). The between-group match of all baseline features was also present when separately assessed in each sex (Table 1). Regarding intervention compliance, 3 individuals withdrew from the IFI intervention due to busy working schedule or dislike of increased urination, while 2 individuals withdrew from the IFI + HUS intervention due to travel or sleep disturbance. These 5 individuals were excluded from the study and the analyses. All included individuals (n = 35) complied with the interventions. The average water intake per day was 46.8 ± 4.8 and 44.5 ± 6.1 mL kg^−1^ in the IFI and IFI + HUS (no difference between interventions, *P* = 0.248). In addition, in the IFI + HUS intervention, the average HR per hour was elevated during the initial hours of sleep compared with supine rest (*P* < 0.001) (Supplemental Figure 1).

**Table 1:** General Characteristics (Demographics, Anthropometrics, Fitness, Body Composition) at Baseline

	IFI			IFI + HUS			*P* Value (IFI Vs IFI + HUS)		
	Female (n = 13)	Male (n = 11)	All (N = 24)	Female (n = 5)	Male (n = 6)	All (N = 11)	Female	Male	All
Sex (female/male)			13/11			5/6			0.725
Age (y)	39.1 ± 18.8	44.8 ± 18.6	41.8 ± 18.5	41.3 ± 18.4	42.1 ± 21.7	41.7 ± 19.3	0.834	0.787	0.993
Height (cm)	160.0 ± 7.6	173.3 ± 5.8	166.1 ± 9.5	159.3 ± 5.6	173.0 ± 4.0	166.7 ± 8.4	0.851	0.884	0.855
Weight (kg)	54.6 ± 10.3	69.4 ± 8.9	61.4 ± 12.1	52.1 ± 7.0	68.2 ± 8.0	60.9 ± 11.1	0.614	0.797	0.905
BMI (kg·m^−2^)	21.2 ± 2.3	23.0 ±0 .0 2	22.0 ± 2.3	20.5 ± 2.0	22.8 ± 2.1	21.7 ± 2.3	0.549	0.815	0.726
BSA (m^2^)	1.56 ± 0.17	1.83 ± 0.14	1.68 ± 0.21	1.52 ± 0.12	1.81 ± 0.11	1.68 ± 0.19	0.663	0.797	0.971
MVPA (hr·wk^−1^)	4.2 ± 3.0	6.5 ± 5.1	5.3 ± 4.2	3.5 ± 3.0	9.5 ± 4.8	6.8 ± 5.0	0.664	0.258	0.357
MVPA endurance (h·wk^−1^)	3.9 ± 3.1	5.0 ± 4.5	4.4 ± 3.8	3.5 ± 3.0	9.0 ± 5.3	6.5 ± 5.1	0.791	0.114	0.177
RPE endurance	10.9 ± 3.9	12.8 ± 1.8	11.8 ± 3.2	8.4 ± 6.1	13.8 ± 1.6	11.4 ± 4.9	0.308	0.274	0.760
Smoking (%)	0	0	0	0	0	0	1.000	1.000	1.000
VO_2peak_ (ml·min^−1^ kg^−1^)	35.2 ± 8.1	44.7 ± 9.6	39.6 ± 9.9	33.6 ± 9.2	43.7 ± 9.6	39.1 ± 10.4	0.723	0.843	0.907
VO_2peak_ (ml·min^−1^ kg LBM^−1^)	51.1 ± 10.1	57.2 ± 10.8	53.9 ± 10.6	44.6 ± 12.5	55.9 ± 10.9	50.8 ± 12.5	0.261	0.820	0.445
Body composition									
BMC (kg)	1.82 ± 0.38	2.34 ± 0.41	2.06 ± 0.47	1.76 ± 0.35	2.51 ± 0.27	2.17 ± 0.49	0.745	0.397	0.543
LBM (kg)	37.2 ± 6.4	53.8 ± 7.2	44.8 ± 10.8	36.3 ± 5.1	52.8 ± 6.3	45.3 ± 10.3	0.787	0.787	0.895
Fat (kg)^a^	16.6 ± 4.6	14.3 ± 3.2	15.5 ± 4.1	15.2 ± 3.0	13.9 ± 3.0	14.5 ± 3.0	0.775	0.733	0.563
Fat (%)	29.5 ± 4.7	20.3 ± 3.6	25.3 ± 6.3	28.5 ± 3.9	19.9 ± 2.9	23.8 ± 5.5	0.661	0.823	0.500

Values are mean ± SD or %.

Number of biological observations for each variable in IFI = 24.

Number of biological observations for each variable in IFI + HUS = 11.

BMC = bone mineral content; BMI = body mass index; BSA = body surface area; HUS = head-up sleep; IFI = increased fluid intake; LBM = lean body mass; MVPA = moderate-to-vigorous physical activity; MVPA endurance = moderate-to-vigorous physical activity comprising endurance exercise; RPE endurance = ratio of perceived exertion of endurance exercise; VO_2peak_ = peak oxygen uptake.

a Statistical tests: independent sample *t*-test or Mann-Whitney *U* test if a variable is not-normally distributed.

### Hemoglobin mass and intravascular volumes

The effects of the intervention on circulating hemoglobin mass, blood O_2_ carrying capacity, and intravascular volumes (red blood cell volume, plasma volume, BV) are presented in Table 2. None of these variables were affected by IFI or IFI + HUS and between-group effects were not observed (*P* ≥ 0.246).

**Table 2:** Hemoglobin Mass, Blood O_2_ Carrying Capacity, and Intravascular Volumes

	IFI		IFI + HUS		ANCOVA		
	Pre	Post	Pre	Post	Group Effect^a^	95% CI	*P* Value
Hb_mass_ (g)	686.0 ± 215.2	680.5 ± 194.7	709.2 ± 219.0	708.8 ± 215.2	+8.1	−45.2 to 61.3	0.760
Hb_mass_ (g·kg^−1^)	11.1 ± 2.2	11.0 ± 1.8	11.5 ± 2.2	11.4 ± 1.8	+0.1	−0.8 to 1.0	0.793
Hb (g·dL^−1^)	13.9 ± 1.7	13.6 ± 1.4	13.4 ± 1.3	13.5 ± 1.3	+0.3	−0.2 to 0.8	0.255
Hct (%)	42.7 ± 5.0	41.8 ± 4.2	40.9 ± 4.0	41.3 ± 4.0	+0.9	−0.6 to 2.3	0.246
RBCV (mL)	2,101 ± 655	2089 ± 595	2,173 ± 671	2,175 ± 659	+23.1	−141.4 to 187.6	0.776
PV (mL)	3,220 ± 619	3,341 ± 627	3,581 ± 763	3,550 ± 829	−97.8	−412.2 to 216.6	0.531
BV (mL)	5,321 ± 1,215	5,430 ± 1,170	5,754 ± 1,399	5,724 ± 1,433	−82.5	−532.5 to 367.5	0.711
RBCV (mL·kg^−1^)	33.9 ± 6.6	33.8 ± 5.6	35.4 ± 6.8	35.1 ± 5.5	+0.3	−2.4 to 3.1	0.798
PV (mL·kg^−1^)	53.0 ± 7.1	54.9 ± 6.8	59.1 ± 7.1	58.2 ± 7.4	+0.4	−4.6 to 5.4	0.869
BV (mL·kg^−1^)	86.9 ± 11.4	88.8 ± 10.4	94.4 ± 12.7	93.4 ± 11.2	+1.0	−6.1 to 8.1	0.775

Values are as mean ± SD, *β* or 95% CI.

Number of biological observations for each variable in IFI = 24.

Number of biological observations for each variable in IFI + HUS = 11.

Statistical tests: ANCOVA.

ANCOVA = analysis of covariance; BV = blood volume; Hb = hemoglobin concentration; Hb_mass_ = circulating hemoglobin mass; Hct = hematocrit; PV = plasma volume; RBCV = red blood cell volume; other abbreviations as in Table 1.

a Mean difference in post variables (ie, post Hb_mass_) in HUS vs control, including baseline (pre) variables as covariates.

### Resting cardiac structure, function and hemodynamics

The effects of the intervention emerged at the volumetric, structural, and functional level in the heart at rest (Table 3). With respect to cardiac volumes, left ventricular end-diastolic volume (LVEDV) and stroke volume (SV) were increased after IFI and IFI + HUS (*P* ≤ 0.007); the effect on SV was larger in IFI + HUS compared with IFI (*P* = 0.037). Regarding biomechanical and structural properties of the heart, arterial elastance (Ea) was similarly reduced after IFI and IFI + HUS (*P* ≤ 0.006). LV diastolic elastance (Ed) was increased after IFI + HUS (*P* = 0.010), with no difference relative to the effect of IFI (*P* = 0.442). LV_mass_ only increased after IFI (*P* < 0.001), although this effect did not differ relative to the effect of IFI + HUS (*P* = 0.951). As for LV function, systolic emptying rate (SER) was increased after IFI and IFI + HUS (*P* ≤ 0.005); this effect was larger in IFI + HUS compared with IFI (*P* = 0.032). Diastolic filling rate, myocardial tissue lateral and septal e’ as well as lateral E/e’ were only increased after IFI + HUS (*P* ≤ 0.049); among these effects, only that in lateral e’ differed in IFI + HUS compared with IFI (*P* = 0.043). Finally, arterial blood pressures were not modified, whereas total peripheral resistance (TPR) was similarly reduced after IFI and IFI + HUS (*P* ≤ 0.038).

**Table 3:** Cardiac Volumes, Mass, Left Ventricular Function, Arterial Pressure, and Peripheral Resistance at Rest

	IFI		IFI + HUS		ANCOVA		
	Pre	Post	Pre	Post	Group Effect^a^	95% CI	*P* Value
Cardiac volumes and output							
RA (mL·m^−2^)	14.3 ± 3.6	14.9 ± 3.5	14.3 ± 4.5	15.9 ± 5.2	+1.0	−0.6, 2.5	0.234
LA (mL·m^−2^)	15.6 ± 3.3	16.5 ± 3.4	15.6 ± 4.0	16.6 ± 5.5	+0.2	−2.0, 2.3	0.862
LVEDV (mL·m^−2^)	71.8 ± 16.3	75.8 ± 15.4∗	74.4 ± 9.1	81.2 ± 12.1∗	+3.0	−1.5, 7.5	0.180
LVESV (mL·m^−2^)	13.7 ± 4.4	14.4 ± 4.5	14.3 ± 3.6	14.4 ± 4.1	−0.2	−3.3, 2.8	0.869
SV (mL·m^−2^)	58.1 ± 13.6	61.4 ± 12.4∗	60.1 ± 8.2	66.8 ± 8.7∗	+3.7	0.2, 7.1	0.037
HR (bpm)	60.7 ± 9.9	59.9 ± 9.7	60.5 ± 11.6	59.4 ± 8.8	−0.4	−6.2, 5.3	0.884
Q (L·min^−1^ m^−2^)	3.5 ± 0.7	3.6 ± 0.7	3.6 ± 0.6	4.0 ± 0.8∗	+0.2	−0.2, 0.6	0.284
LV mass and stiffness							
LV_mass_ (g·m^−2^)	70.3 ± 17.0	74.4 ± 18.2∗	75.8 ± 22.3	79.9 ± 24.0	−0.1	−4.2, 4.0	0.951
Ees (mm Hg·mL^−1^ m^2^)	8.79 ± 3.65	7.91 ± 2.26	8.09 ± 2.57	7.96 ± 2.15	+0.2	−1.3, 1.8	0.762
Ed (m^2^·mL^−1^)	0.09 ± 0.03	0.09 ± 0.02	0.08 ± 0.02	0.09 ± 0.03∗	+0.01	−0.01, 0.02	0.442
Ea (mm Hg·mL^−1^ m^2^)	1.91 ± 0.40	1.76 ± 0.34∗	1.81 ± 0.16	1.62 ± 0.21∗	−0.1	−0.2, 0.1	0.373
LV function							
DFR (mL·s^−1^ m^−2^)	109.5 ± 28.7	117.5 ± 31.2	96.5 ± 20.3	121.3 ± 29.8∗	+10.8	−10.3, 31.9	0.304
SER (mL·s^−1^ m^−2^)	172.1 ± 54.8	199.3 ± 45.4∗	162.1 ± 26.0	218.9 ± 38.0∗	+25.9	2.3, 49.5	0.032
Mitral E (cm·s^−1^)	75.4 ± 16.2	80.6 ± 17.8	70.7 ± 15.6	84.6 ± 20.0	+7.3	−3.7, 18.4	0.186
Mitral A (cm·s^−1^)	49.3 ± 13.1	52.8 ± 12.7	47.9 ± 12.5	52.3 ± 13.5	+0.3	−7.6, 8.2	0.936
Mitral E/A	1.6 ± 0.5	1.6 ± 0.6	1.6 ± 0.5	1.7 ± 0.7	+0.2	−0.2, 0.5	0.292
Lateral e’ (cm·s^−1^)	17.7 ± 3.3	16.9 ± 3.2	15.6 ± 4.0	17.5 ± 4.4∗	+2.0	0.1, 3.9	0.043
Septal e’ (cm·s^−1^)	14.5 ± 2.7	15.0 ± 2.6	13.4 ± 2.5	14.6 ± 2.9∗	+0.5	−0.7, 1.6	0.431
Lateral E/e’	4.3 ± 1.1	4.8 ± 0.9	4.6 ± 1.0	5.0 ± 1.3∗	+0.04	−0.66, 0.73	0.917
Septal E/e’	5.3 ± 1.2	5.4 ± 1.1	5.4 ± 1.3	6.0 ± 1.8	+0.5	−0.2, 1.2	0.158
Arterial pressure and peripheral resistance							
SAP (mm Hg)	119.1 ± 14.9	116.2 ± 10.7	119.7 ± 9.5	118.6 ± 8.9	+2.1	−3.4, 7.6	0.442
DAP (mm Hg)	75.0 ± 11.4	73.0 ± 8.5	75.5 ± 9.7	72.7 ± 8.3	−0.5	−5.3, 4.3	0.828
MAP (mm Hg)	89.7 ± 11.7	86.6 ± 8.5	90.2 ± 8.6	87.2 ± 7.2	+0.3	−3.9, 4.6	0.881
TPR (dyn·s·cm^−5^)	1,299.8 ± 358.5	1,195.0 ± 291.3∗	1,243.2 ± 294.6	1,108.9 ± 298.4∗	−48.5	−189.8, 92.9	0.490

Values are as mean ± SD, *β* or 95% CI.

Number of biological observations for each variable in IFI = 24.

Number of biological observations for each variable in IFI + HUS = 11.

Statistical tests: ANCOVA.

DFR = diastolic filling rate; Ea = effective arterial elastance; Ed = diastolic elastance; Ees = end-systolic elastance; e’ = tissue myocardial velocity in early diastole (in the lateral or septal wall); E/e’ ratio = ratio of peak blood flow velocity to tissue myocardial velocity in early diastole; DAP = diastolic arterial pressure; HR = heart rate; LVEDV = left ventricular end-diastolic volume; LVESV = left ventricular end-systolic volume; LV = left ventricle; LV_mass_ = left ventricular mass; MAP = mean arterial pressure; Mitral E/A = ratio of peak blood flow velocity in early diastole (mitral E wave) to peak blood flow velocity in late diastole due to atrial contraction (mitral A wave); Q = cardiac output; RA = right atrial volume; SAP = systolic arterial pressure; SER = systolic emptying rate; SV = stroke volume; TPR = total peripheral resistance; LA = left atrial volume; other abbreviations as in Table 1.

∗ *P* < 0.05, post vs pre.

a Mean difference in post variables (ie, post RA) in IFI + HUS vs IFI, including baseline (pre) variables as covariates.

### Exercise cardiac structure and function

**Figure 1:**
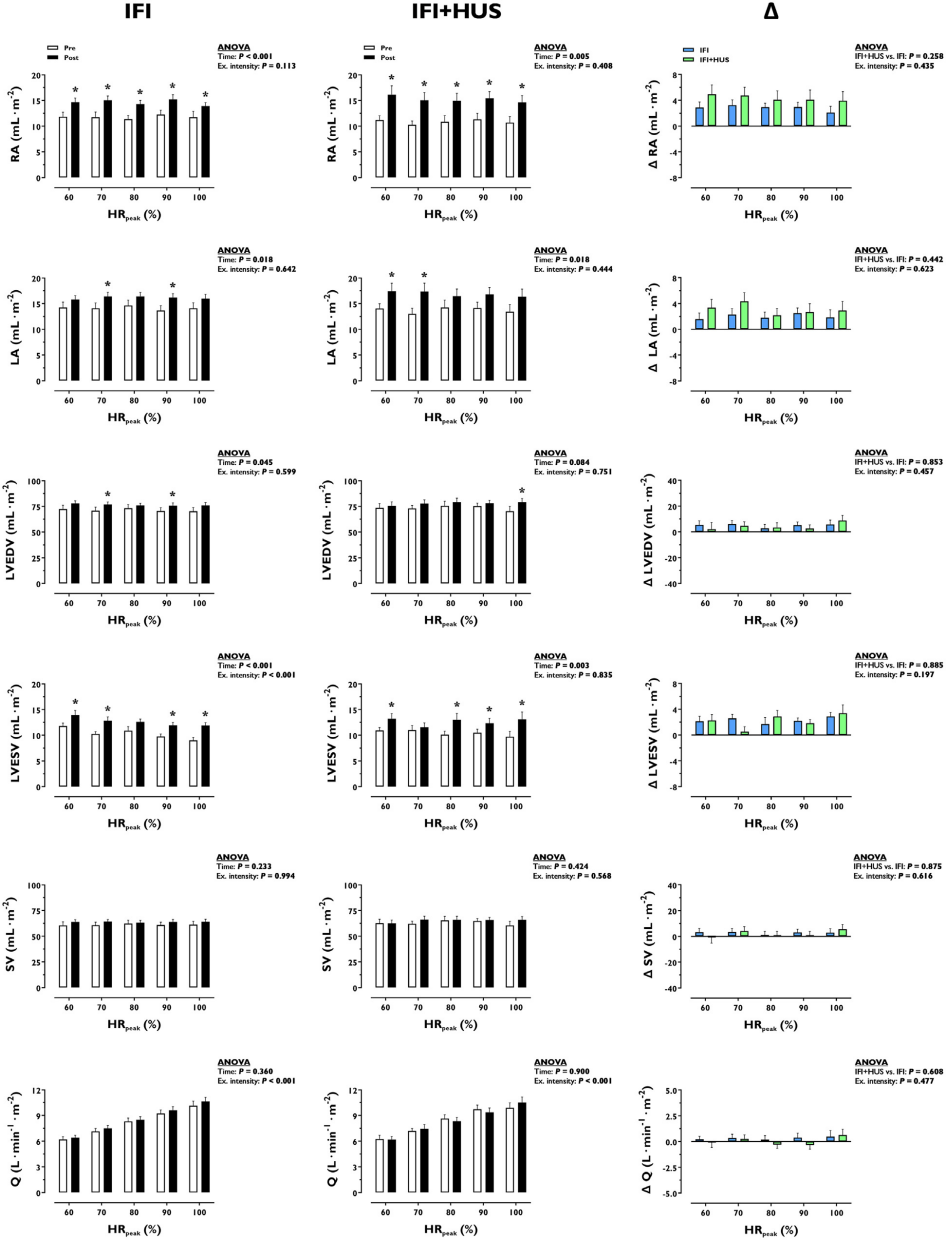
**Effect of Increased Fluid Intake or Increased Fluid Intake + Head-Up Sleep on Cardiac Volumes and Output during Moderate to Peak Exercise** Data are illustrated as mean ± SEM. ∗*P* < 0.05, post vs. pre, within each group at a specific exercise intensity. Δ, IFI + HUS minus IFI. Number of biological observations for each graph in IFI = 240. Number of biological observations for each graph in IFI + HUS = 110. Number of biological observations for each graph in Δ = 175. Statistical tests: 2-way ANOVA with repeated measures with time (or group) and exercise intensity as factors; dependent sample *t*-test. HR_peak_ = peak heart rate; LA = left atrial volume; LVEDV = left ventricular end-diastolic volume; LVESV = left ventricular end-systolic volume; Q = cardiac output; RA = right atrial volume; SV = stroke volume; IFI = increased fluid intake; IFI + HUS = increased fluid intake plus head-up sleep.

Cardiac volumes and output during moderate to peak exercise are presented in Figure 1. Right and left atria were enlarged during exercise after IFI and IFI + HUS (*P* ≤ 0.018). LVEDV during exercise was augmented after IFI (*P* = 0.045), not after IFI + HUS (*P* = 0.084). Both IFI and IFI + HUS increased left ventricular end-systolic volume during exercise (*P* = 0.003). Accordingly, neither SV nor cardiac output (Q) during exercise were altered by IFI or IFI + HUS (*P* ≥ 0.360). At a moderate absolute exercise intensity (100 W), HR was reduced by IFI (125.4 ± 14.9 vs 117.5 ± 17.7 beats/min, *P* = 0.004) and IFI + HUS (126.2 ± 17.0 vs 117.2 ± 23.0 beats/min, *P* = 0.034). None of the effects on exercise cardiac variables differed between interventions (*P* ≥ 0.258).

### Exercise O_2_ extraction and consumption

**Figure 2:**
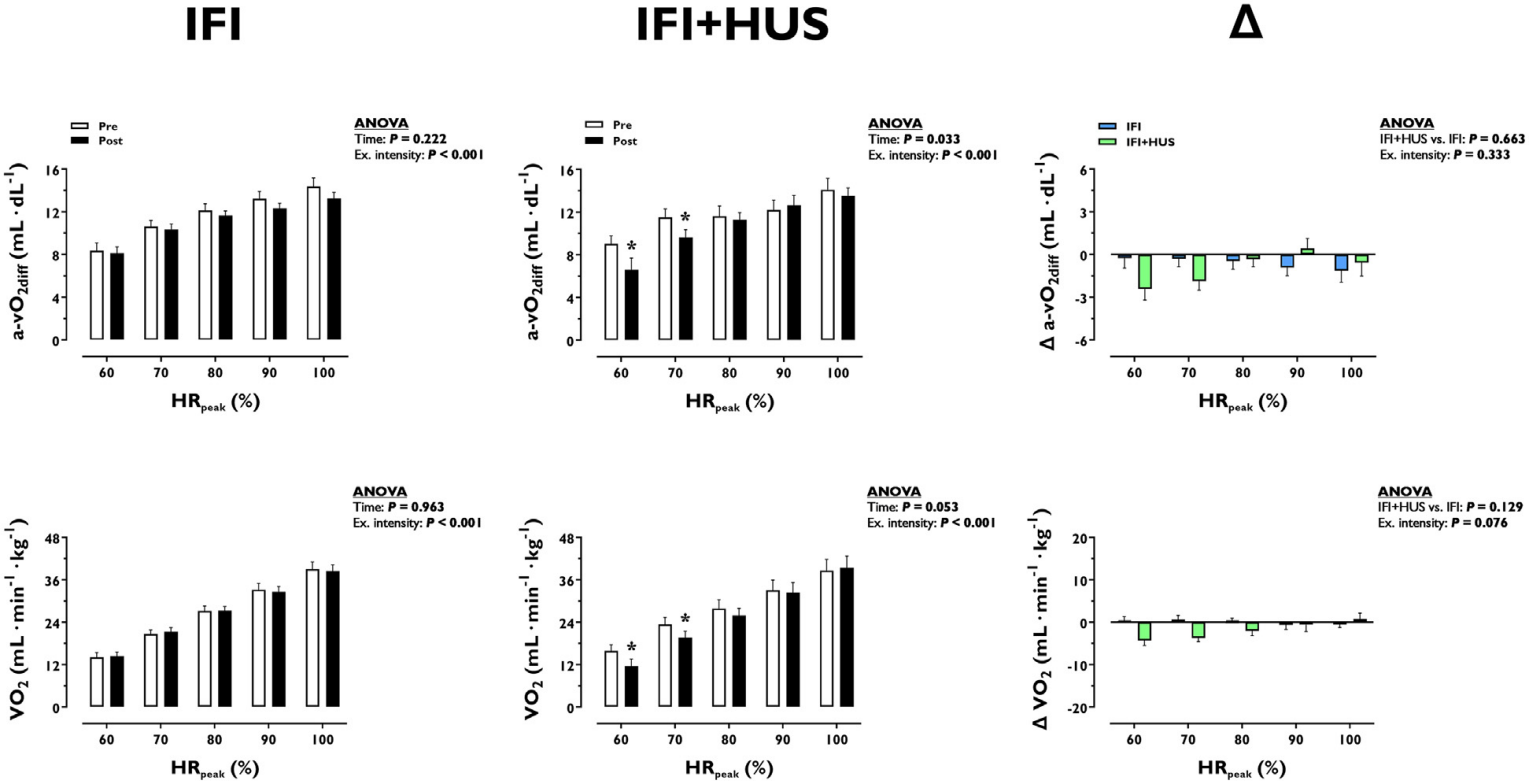
**Effect of Increased Fluid Intake or Increased Fluid Intake + Head-Up Sleep on Whole-Body O_2_ Extraction and Consumption During Moderate to Peak Exercise** Data are illustrated as mean ± SEM. ∗*P* < 0.05, post vs. pre, within each group at a specific exercise intensity. Δ, IFI + HUS minus IFI. Number of biological observations for each graph in IFI = 240. Number of biological observations for each graph in IFI + HUS = 110. Number of biological observations for each graph in Δ = 175. Statistical tests: 2-way ANOVA with repeated measures with time (or group) and exercise intensity as factors; dependent sample *t*-test. a-vO_2diff_ = arteriovenous O_2_ difference; HR_peak_ = peak heart rate; VO_2_ = oxygen cconsumption; other abbreviations as in Figure 1.

Figure 2 shows the effects of the intervention on whole-body O_2_ extraction (a-vO_2diff_) and consumption (VO_2_) during moderate to peak exercise. Neither a-vO_2diff_ nor VO_2_ during exercise were modified after IFI (*P* ≥ 0.222), whereas a-vO_2diff_ was reduced (*P* = 0.033) but not VO_2_ (*P* = 0.053) after IFI + HUS. The effects on a-vO_2diff_ and VO_2_ during exercise did not differ between interventions (*P* ≥ 0.129).

## Discussion

This study determined the medium-term (3-month) effects of IFI or IFI plus HUS (IFI + HUS) on the resting and stressed cardiorespiratory phenotype, comprising strong predictors of overall and cardiovascular mortality. The main findings are i) IFI or IFI + HUS do not alter intravascular volumes, blood O_2_ carrying capacity, cardiac or aerobic capacities; ii) resting cardiac volumes (specifically LVEDV and SV) are increased and cardiac afterload decreased by IFI and IFI + HUS, the latter eliciting a greater volumetric effect; iii) LV diastolic function is exclusively increased by IFI + HUS.

The postulated mechanistic rationale underpinning the present investigation primarily depended on the expansion of BV.^7^^,^^8^ In contrast to prior observations in a sample of 12 patients with vasovagal symptom,^11^ BV was not increased by the interventions. In fact, intravascular volumes and circulating hemoglobin mass remained remarkably stable following IFI and IFI + HUS (Table 2), concurring with a tight regulation of body fluids and blood O_2_ carrying capacity in healthy individuals.^8^ Accordingly, this study demonstrates that the homeostatic balance of intravascular volumes and blood O_2_ carrying capacity in the healthy human body is robust to a large daily increment (+40%) in water intake and concomitant stimulation of BV-regulating hormones during the night. In the presence of a deficit in and/or altered regulation of BV, the medium-term interventions implemented in this study might have elicited BV expansion and subsequently improved circulatory function.^11^ Indeed, HUS, as a regular therapy, may improve orthostatic tolerance, albeit with a variable degree of effectiveness, in patients with vasovagal syncope, orthostatic hypotension, and postural orthostatic tachycardia syndrome.^11^^,^^22^ The combination of IFI + HUS might provide further stimuli to induce beneficial adaptions in orthostatic tolerance.^23^ Nonetheless, caution should be taken to implement these interventions in patients presenting with orthostatic intolerance and hypervolemia, for example, decompensated heart failure patients, until specific evidence in this population becomes available. Notwithstanding, IFI and IFI + HUS did not alter BV but elicit circulatory improvements in healthy individuals, as discussed hereunder.

The filling of the heart is primarily determined by BV, as consistently demonstrated by blood withdrawal and intravascular infusion studies.^7^^,^^24^ Despite the unchanged BV, IFI and IFI + HUS elicited structural and functional adaptations in the LV, markedly observed at rest. Both interventions increased the maximum volumetric size of the LV during the relaxation period (diastole) and increased the rate of blood delivered by the LV per beat (SER). These volumetric adaptations were accompanied by reduced cardiac afterload, as reflected by reduced arterial elastance and TPR. Hence, the resistance to perfuse the systemic circulation was attenuated, concomitant to increased cardiac filling. What combination of stimuli, seemingly independent of BV, underlay the present medium-term adaptations? In this regard, the reduction of TPR via vasodilatory stimuli is known to increase Q in the absence of BV alterations.^25^ In both interventions, arterial blood pressures at rest were seemingly decreased—the study was not designed to detect small changes in BP—, possibly contributing to reduced TPR and arterial elastance. Thus, the common experimental intervention, that is, increased water intake, might have a small antihypertensive effect, concurring with recent epidemiological findings in our study population.^26^ Moreover, BV is partly regulated by mechanoreceptors located in atrial and ventricular walls that sense the filling of the heart.^27^^,^^28^ In response to increase cardiac filling, natriuretic peptides are released from cardiac walls into the circulation leading to: 1) augmented renal excretion of sodium and water, thereby decreasing intravascular (plasma) volume, as well as 2) reduced TPR due to direct stimulation of peripheral vasodilation.^27^^,^^29^ Indeed, if medium-term hemodynamic (exercise) stimuli acutely augmenting heart filling pressures exceed a certain threshold, BV is unaltered but LV filling and Q are increased.^30^^,^^31^ Collectively considered, medium-term increased water intake might have acutely increased heart filling pressures, stimulating cardiac mechanoreceptors to trigger the natriuretic feedback mechanism to such an extent that BV was preserved. While further studies are necessary to determine the ultimate explanation, both IFI and IFI + HUS improved resting circulatory variables, notably arterial elastance and TPR, having a potential prognostic impact that cannot be elucidated by the present medium-term study design.^32^^,^^33^

The cardiac capacity to deliver blood to active muscles defines the “fitness” of the circulatory system.^34^ Neither IFI nor IFI + HUS enhanced peak Q, albeit the largest volume of the LV (LVEDV) at high exercise intensities was enhanced by both interventions (Figure 1). This was explained by decreased LV emptying during exercise, as reflected by increased left ventricular end-systolic volume, which resulted in unaltered SV. Consequently, the enhancing effect of the interventions on the filling of the LV was not accompanied by a greater overall LV contractility during exercise, as could be expected conforming to the Frank-Starling mechanism.^6^ Given that peak Q and blood O_2_ carrying capacity were unaltered by the interventions, peak O_2_ delivery and consumption (VO_2peak_) remained stable. These findings indicate that in the absence of additional medium-term hemodynamic stimuli (other than increased cardiac filling), the circulatory system does not adapt its capacity to deliver blood and O_2_ to the periphery, which are essential to enhance cardiorespiratory fitness.^34^

The addition of HUS elicited some quantitatively and qualitatively distinct adaptations (Central Illustration). IFI + HUS induced greater effects on resting SV and SER compared with IFI. These results suggest a larger impact of IFI + HUS on LV contractility at rest —considering the fact that the resistance encountered by the LV to eject blood, as indicated by the effects on arterial elastance and TPR, was similarly reduced at rest by IFI and IFI + HUS. In this respect, HUS may entail adaptations in LV contractility in response to the HUS-dependent increase in TPR and cardiac afterload.^35^ In the relaxation phase of the heart (diastole), adaptations were only observed following IFI + HUS. Namely, myocardial tissue lateral e’, an established prognostic factor which reflects the velocity of the LV lateral wall in the early phase of relaxation,^36^ was increased after IFI + HUS. Conceivably, the stimuli of reduced venous return provided by HUS may have induced counteracting adaptations aiming to enhance diastolic function and thereby cardiac filling.

**Central Illustration:**
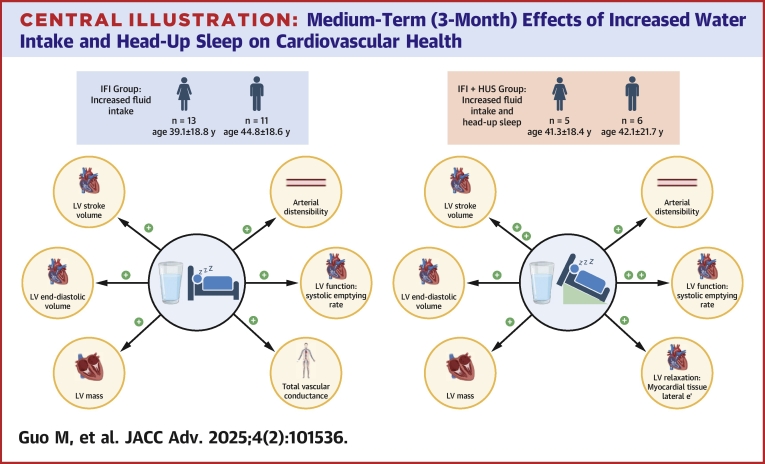
**Medium-Term (3-Month) Effects of Increased Water Intake and Head-Up Sleep on Cardiovascular Health** LV = left ventricle; other abbreviations as in Figure 1.

### Study Limitations

A control intervention for time was not included due to long-established evidence demonstrating stable hematological and cardiac phenotypes over 3 months in our study population.^37^, ^38^, ^39^, ^40^ In other respects, the study was conducted in healthy individuals mainly comprising the adult lifespan, including a balanced number of women and men, in order to avoid the influence of confounding sex-related, pathophysiological, and/or pharmacological factors; hence, the findings can plausibly be applicable to the majority of the population. Yet, whether the findings can be extrapolated to clinical conditions with altered hematological and cardiorespiratory phenotypes remains to be elucidated.

## Conclusions

This study discloses that a 3-month lifestyle intervention augmenting water intake expands the volume and output of the LV and reduces arterial elastance and cardiac afterload, without altering intravascular volumes, cardiac or aerobic capacities in healthy adults throughout the adult lifespan, irrespective of sex. With the addition of HUS, relaxation properties of the LV at rest are further improved. Future studies may determine the prognostic impact of such a sustained lifestyle-induced modification of cardiac variables associated with mortality and life expectancy.

Perspectives **COMPETENCY IN MEDICAL KNOWLEDGE:** Whether increased water intake alone or in combination with nonexercise interventions such as HUS, aimed to expand BV, improve the human cardiovascular phenotype and cardiorespiratory fitness remains unknown.

**TRANSLATIONAL OUTLOOK:** Medium-term (3 months) increased water intake expands the volume and output of the heart and reduces arterial elastance and cardiac afterload, without altering intravascular volumes, cardiac or aerobic capacities, in women and men irrespective of sex. The addition of HUS enhances relaxation properties of the heart at rest.

## Funding support and author disclosures

This work was supported by Research Grants Council of Hong Kong-Early Career Scheme (106210224, to Dr Montero) and Seed Fund (104006024, to Dr Montero). The authors have reported that they have no relationships relevant to the contents of this paper to disclose.
