# Supplementary material for: Medium-Term Effects of Increased Water Intake and Head-Up Sleep on Cardiovascular Health
Source: JACC Adv. 2025 Jan 8;4(2):101536. doi: 10.1016/j.jacadv.2024.101536 (PMC11780084; doi:10.1016/j.jacadv.2024.101536)
Supplement: Supplemental_Material [file mmc1.docx]

**SUPPLEMENTAL APPENDIX**

**Detailed description of measurements**

*Cardiac structure, stiffness, function and hemodynamics.* Apical four-chamber and two-chamber cine-loops were recorded via high-resolution ultrasound (Mindray Medical M9) at rest and continuously (sequentially) throughout an established incremental exercise test (detailed in the subsequent ‘Aerobic capacity’ section) in a cycle ergometer (KICKR Core, Wahoo) setup designed to facilitate maximal effort and precise echocardiography, as previously described [^1-4^](#_ENREF_1). Following the American Society of Echocardiography and the European Association of Cardiovascular Imaging recommendations, cardiac chamber quantification was performed offline using the modified Simpson method (biplane method of disks) by tracing the endocardial border of the left ventricle (LV) in both apical four-chamber and two-chamber views at end-diastole and end-systole [^5^](#_ENREF_5)^,^[^6^](#_ENREF_6). Stroke volume (SV) was determined as left ventricular end-diastolic volume (LVEDV) minus LV end-systolic volume (LVESV), while the product of SV and heart rate (HR) provided cardiac output (Q). The recommended Cube algorithm was used to quantify LV_mass_ [^5^](#_ENREF_5). LV relative wall thickness (LVRWT) was determined according to the equation: LVRWT = 2 × LV posterior wall thickness at end-diastole (LVPWd) / LV internal diameter at end-diastole (LVIDd). With respect to cardiac stiffness, LV end-systolic elastance (Ees = systolic blood pressure (SBP) / LVESV), LV diastolic elastance (Ed = E/e’ / LV SV) and effective arterial elastance (Ea = SBP / LV SV) were determined conforming to consensus recommendations [^7^](#_ENREF_7)^,^[^8^](#_ENREF_8). Right (RA) and left atrial (LA) volumes were measured using the single plane Simpson’s method of disks from the apical four-chamber view at end-systole [^5^](#_ENREF_5)^,^[^9^](#_ENREF_9). RV dimensions were not analyzed due to the uncertain imaging quality of such a morphologically complex chamber [^10^](#_ENREF_10). Regarding the assessment of LV function, transmitral inflow velocities were recorded via pulsed-wave Doppler, with the sample volume placed between the mitral leaflet tips in the apical four-chamber view. The peak inflow velocities during early (E) and late (A) diastole and the ratio (E/A) was determined. Likewise, myocardial tissue e’ and a’ velocities at the lateral and septal walls were recorded via TDI and the E/e’ ratio was calculated [^5^](#_ENREF_5). Blood pressures, comprising SBP, diastolic (DBP) and mean arterial pressure (MAP), were assessed at rest in the upper arm via the gold-standard auscultatory method using a mercury sphygmomanometer. Total peripheral resistance (TPR) was determined as the ratio of MAP and Q.

*Aerobic capacity.* Pulmonary O_2_ uptake (VO_2_), CO_2_ output and ventilation were recorded via a mixing chamber system (KORR Medical, USA) throughout the incremental exercise test. Following a warm-up period at 10-30 W, the workload was progressively increased by 10-30 W increments every 50 s until exhaustion was reached in the recommended total duration of 7-9 min [^11^](#_ENREF_11). Calibration of the gas analyzers and the flowmeter was conducted prior to each test. Values were averaged over 15 s following current recommendations [^12^](#_ENREF_12). The highest average value defined VO_2peak_ provided that at least two of the following established criteria were fulfilled: (i) plateau in O_2_ uptake despite increased workload, (ii) age- and body position-predicted HR_peak_ +/- 10 bpm [^13^](#_ENREF_13)^,^[^14^](#_ENREF_14) and/or (iii) respiratory exchange ratio (RER) > 1 [^15^](#_ENREF_15). VO_2peak_ was expressed relative to kg of body weight or lean body mass (LBM), the latter normalization accurately reflecting fitness status independently of sex [^1^](#_ENREF_1)^,^[^16^](#_ENREF_16). The arterio-venous O_2_ difference (a-vO_2diff_) during incremental exercise was determined by the Fick Principle (VO_2_ = LV Q × a-vO_2 diff_).

*Intravascular volumes.* Red blood cell volume (RBCV), plasma volume (PV) and total BV were determined from the primary measurement of circulating hemoglobin mass (Hb_mass_) via the classic carbon monoxide (CO) rebreathing technique integrated in a semi-automated system with a low typical error of measurement (TE ≤ 1.2 %), as previously detailed [^17^](#_ENREF_17)^,^[^18^](#_ENREF_18). This validated method has been shown to precisely identify minor decrements in Hb_mass_ (-3 %, 26-28 g) induced by phlebotomy in healthy young individuals [^19^](#_ENREF_19). In brief, 2 mL of blood were sampled at rest from the cannulated antecubital vein and analysed immediately in duplicate for percent carboxyhemoglobin (%HbCO), hemoglobin (Hb) concentration and hematocrit (Hct) (ABL80, Radiometer) in both testing visits. Then, the participants breathed 100 % O_2_ plus a bolus of 1.5 mL/kg of 99.5 % chemically pure CO (CO H.P. Grade, SG, Hong Kong) in a closed breathing circuit for 10 min. An additional 2 mL blood sample was obtained exactly at min 10 of CO-rebreathing and analysed in duplicate as aforementioned. The change in %HbCO induced by CO administration was used to calculate circulating hemoglobin mass (Hb_mass_), taking into account the small amount of CO that remains in the rebreathing circuit at the end of the procedure. Hb_mass_, along with rest pre-infusion Hb concentration and Hct, comprise the required variables to determine RBCV, PV and BV [^17^](#_ENREF_17)^,^[^18^](#_ENREF_18).

*Body composition*. Body composition was assessed via dual-energy x-ray absorptiometry (DXA) (Hologic QDR 4500; Hologic, Inc) according to current recommendations [^20^](#_ENREF_20). In brief, the participants were instructed to sit down in the middle of the scanning table and subsequently lay supine with their spine aligned with the printed longitudinal midline. Once they were in the supine position, their arms were placed along their side with the palms pronated to standardize the scanned area for all individuals. Bone mineral content, LBM and fat body mass were determined.

**Supplemental Figure 1.** Average heart rate (HR) per hour during sleep (8 hr) throughout the IFI+HUS intervention compared with HR at supine rest.

Number of biological observations = 102.

Statistical tests: none.

**REFERENCES**

1. Diaz-Canestro C, Pentz B, Sehgal A, Montero D. Sex dimorphism in cardiac and aerobic capacities: The influence of body composition. *Obesity (Silver Spring).* 2021;29(11):1749-1759.

2. Diaz-Canestro C, Pentz B, Sehgal A, Montero D. Sex Differences In Cardiorespiratory Fitness Are Explained By Blood Volume And Oxygen Carrying Capacity. *Cardiovasc Res.* 2021.

3. Diaz-Canestro C, Pentz B, Sehgal A, Yang R, Xu A, Montero D. Lean body mass and the cardiovascular system constitute a female-specific relationship. *Sci Transl Med.* 2022;14(667):eabo2641.

4. Pugliese NR, N DEB, Balletti A, et al. Characterization of hemodynamic and metabolic abnormalities in the heart failure spectrum: the role of combined cardiopulmonary and exercise echocardiography stress test. *Minerva Cardiol Angiol.* 2022;70(3):370-384.

5. Lang RM, Badano LP, Mor-Avi V, et al. Recommendations for cardiac chamber quantification by echocardiography in adults: an update from the American Society of Echocardiography and the European Association of Cardiovascular Imaging. *Eur Heart J Cardiovasc Imaging.* 2015;16(3):233-270.

6. Pellikka PA, Nagueh SF, Elhendy AA, Kuehl CA, Sawada SG, American Society of E. American Society of Echocardiography recommendations for performance, interpretation, and application of stress echocardiography. *J Am Soc Echocardiogr.* 2007;20(9):1021-1041.

7. Ikonomidis I, Aboyans V, Blacher J, et al. The role of ventricular-arterial coupling in cardiac disease and heart failure: assessment, clinical implications and therapeutic interventions. A consensus document of the European Society of Cardiology Working Group on Aorta & Peripheral Vascular Diseases, European Association of Cardiovascular Imaging, and Heart Failure Association. *Eur J Heart Fail.* 2019;21(4):402-424.

8. Redfield MM, Jacobsen SJ, Borlaug BA, Rodeheffer RJ, Kass DA. Age- and gender-related ventricular-vascular stiffening: a community-based study. *Circulation.* 2005;112(15):2254-2262.

9. Ebtia M, Murphy D, Gin K, et al. Best method for right atrial volume assessment by two-dimensional echocardiography: validation with magnetic resonance imaging. *Echocardiography.* 2015;32(5):734-739.

10. Mertens LL, Friedberg MK. Imaging the right ventricle--current state of the art. *Nat Rev Cardiol.* 2010;7(10):551-563.

11. Astorino TA, Rietschel JC, Tam PA, et al. Reinvestigation of optimal duration of VO2max testing. *Journal of Exercise Physiology.* 2004;7(6):1-8.

12. Martin-Rincon M, Calbet JAL. Progress Update and Challenges on VO2max Testing and Interpretation. *Front Physiol.* 2020;11:1070.

13. Nes BM, Janszky I, Wisloff U, Stoylen A, Karlsen T. Age-predicted maximal heart rate in healthy subjects: The HUNT fitness study. *Scand J Med Sci Sports.* 2013;23(6):697-704.

14. Dillon HT, Dausin C, Claessen G, et al. The effect of posture on maximal oxygen uptake in active healthy individuals. *Eur J Appl Physiol.* 2021;121(5):1487-1498.

15. American Thoracic S, American College of Chest P. ATS/ACCP Statement on cardiopulmonary exercise testing. *Am J Respir Crit Care Med.* 2003;167(2):211-277.

16. Tarnopolsky MA. Sex differences in exercise metabolism and the role of 17-beta estradiol. *Med Sci Sports Exerc.* 2008;40(4):648-654.

17. Siebenmann C, Keiser S, Robach P, Lundby C. CORP: The assessment of total hemoglobin mass by carbon monoxide rebreathing. *J Appl Physiol (1985).* 2017;123(3):645-654.

18. Diaz-Canestro C, Pentz B, Sehgal A, Montero D. Sex differences in cardiorespiratory fitness are explained by blood volume and oxygen carrying capacity. *Cardiovasc Res.* 2022;118(1):334-343.

19. Keiser S, Meinild-Lundby AK, Steiner T, et al. Detection of blood volumes and haemoglobin mass by means of CO re-breathing and indocyanine green and sodium fluorescein injections. *Scand J Clin Lab Invest.* 2017;77(3):164-174.

20. Lewiecki EM, Binkley N, Morgan SL, et al. Best Practices for Dual-Energy X-ray Absorptiometry Measurement and Reporting: International Society for Clinical Densitometry Guidance. *J Clin Densitom.* 2016;19(2):127-140.
